# Effect of introducing biologics to patients with rheumatoid arthritis on the risk of venous thromboembolism: a nationwide cohort study

**DOI:** 10.1038/s41598-021-96508-z

**Published:** 2021-08-23

**Authors:** Chao-Ping Chen, Pei-Tseng Kung, Wen-Yu Chou, Wen-Chen Tsai

**Affiliations:** 1 Department of Health Services Administration, China Medical University, No. 100, Sec. 1, Jingmao Rd., Beitun Dist., Taichung, 406040 Taiwan; 2 Department of Orthopaedics, Taichung Veterans General Hospital, Taichung, 40705 Taiwan; 3 Department of Acupressure Technology, Jen-Teh Junior College of Medicine, Nursing and Management, Miaoli, 35664 Taiwan; 4 Department of Healthcare Administration, Asia University, Taichung, 41354 Taiwan; 5 Department of Medical Research, China Medical University Hospital, China Medical University, Taichung, 40402 Taiwan

**Keywords:** Rheumatology, Risk factors

## Abstract

In the United States, 100,000–300,000 patients die from venous thromboembolism (VTE) each year, with more than 500,000 people related hospitalizations. While in Europe, 500,000 people die from VTE each year. Patients with rheumatoid arthritis are at increased risk of VTE. The use of biologics in patients with rheumatoid arthritis may be associated with an increased risk of VTE. We identified all patients who had been newly approved for Catastrophic Illness Card of rheumatoid arthritis extracted the claims data from the National Health Insurance research database and Registry for Catastrophic Illness Patient Database from 2003 to 2016. VTE was defined as the presence of inpatient VTE diagnostic codes (including DVT or PE) according to the discharge diagnosis protocol. An analysis of VTE variables indicated that the incidence of VTE in the biologic group (14.33/10,000 person-years) was higher than that in the conventional drug group (12.61/10,000 person-years). As assessed by the Cox proportional hazards model, the relative HR for VTE in the biologic group (HR: 1.11; 95% CI 0.79–1.55) versus that in the conventional drug group did not reach a significant difference. In conclusion, this study found no significant differences in risk were observed between the use of conventional DMARDs and biologics.

## Introduction

Deep vein thrombosis (DVT) is the formation of a blood clot in a deep vein, primarily occurring in the leg, which can lead to permanent damage. Pulmonary embolism (PE) is a potentially life-threatening condition that occurs in the lungs and causes sudden death in 25% of cases^1–3^. DVT and PE occurring together constitute venous thromboembolism (VTE). In the United States, 100,000 to 300,000 patients die from VTE each year, with more than 500,000 people related hospitalizations^4–6^. While in Europe, 500,000 people die from VTE each year^7^.

Patients with rheumatoid arthritis (RA) are at increased risk of VTE^8,9^. Several cohort studies in the United States, the United Kingdom, Sweden, and Taiwan have shown a significant association between rheumatoid arthritis and VTE^10–15^. However, previous studies have suggested that disease-modifying anti-rheumatic drugs (DMARDs) pose different risks of VTE. According to the study by the American College of Rheumatology (ACR) and the Association of Rheumatology Health Professionals (ARHP) presented at their 2013 meeting, the initiation of biologic drugs in patients with RA was associated with a 2.5-fold increased risk of VTE in the first 180 days^16^. In contrast, a cohort study conducted by the British Society for Rheumatology Biologics Registers (BSRBR) showed no significant association between tumor necrosis factor alpha (TNF-α) inhibitor treatment and VTE in patients with RA^17^.

Research on the association between DMARDs and VTE is limited. Studies on the association between biologics and VTE are scarce. We conducted a nationwide cohort study to analyze the risk and factors associated with VTE in patients with rheumatoid arthritis using different DMARDs in Taiwan.

## Results

### Patient selection

**Figure 1:**
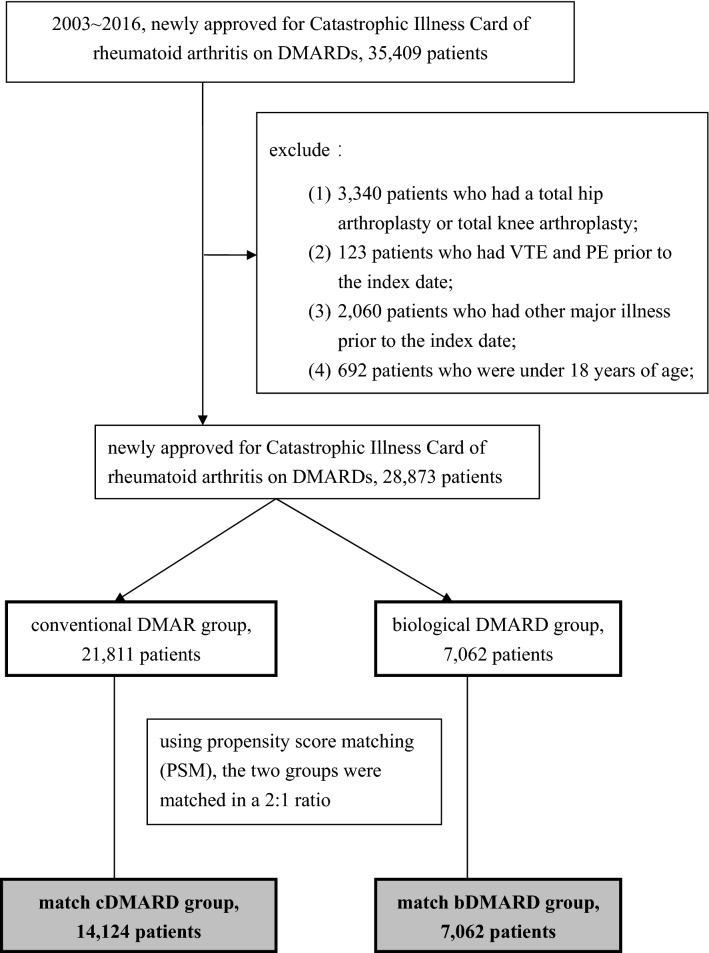
Flowchart showed the identification process of rheumatoid arthritis (RA) for the present study from NHIRD (data extracted from 1 January 2003 to 31 December 2016) using either conventional DMARD or biological DMARD.

The study identified 35,409 patients with newly approved for Catastrophic Illness Card of rheumatoid arthritis using DMARDs from 2003 to 2016, and excluded: (1) 3,340 patients who had a total hip replacement or total knee replacement; (2) 123 patients who had VTE and PE prior to the index date; (3) 2060 patients who had other major injuries prior to the index date; (4) 692 patients who were under 18 years of age; and (5) 321 patients with incomplete data. (Fig. 1).

### Demographic data

A total of 28,873 patients with rheumatoid arthritis were enrolled: 21,811 in the conventional drug group and 7062 in the biologic group (Table 1). In terms of sex and age, the majority of patients in both groups were female and the majority of patients in the conventional drug group were aged 45–54, while the majority of patients in the biologic group were aged 55–64. In respect of insured salary, both groups were prone to be in the range of NT$20,009–22,800. As for the CCI, the majority of patients in both groups had a score of 0, followed by a score of 1. In addition, most patients in both groups had no history of hypertension; had attended medical centers, primarily non-public hospitals and facilities; and tended to receive treatment from high-volume physicians.

**Table 1:** Characteristics of RA patients using different DMARDs.

Variables	Total		cDMARD		bDMARD		*p* Value
	N	%	N	%	NN	%	
Total number	28,873	100.00	21,811	75.54	7062	24.46	
**Sex**							0.001
Male	6979	24.17	5374	24.64	1605	22.73	
Female	21,894	75.83	16,437	75.36	5457	77.27	
**Age (years)**							< 0.001
18–34	2571	8.90	1961	8.99	610	8.64	
35–44	4452	15.42	3477	15.94	975	13.81	
45–54	7790	26.98	5873	26.93	1917	27.15	
55–64	7438	25.76	5376	24.65	2062	29.20	
65–74	4306	14.91	3219	14.76	1087	15.39	
75–100	2316	8.02	1905	8.73	411	5.82	
Mean age (y ± sd)	54.06 ± 14.01		54.10 ± 14.24		53.95 ± 13.26		0.413
**Month salary (NT dollars)**							< 0.001
≦ 20,008	4813	16.67	4173	19.13	640	9.06	
20,009–22,800	8112	28.10	5996	27.49	2116	29.96	
22,801–28,800	6156	21.32	4454	20.42	1702	24.10	
28,801–36,300	2914	10.09	2085	9.56	829	11.74	
36,301–45,800	3449	11.95	2497	11.45	952	13.48	
≧ 45,801	3429	11.88	2606	11.95	823	11.65	
**Urbanization**							0.067
Level 1	8603	29.80	6552	30.04	2051	29.04	
Level 2	8966	31.05	6831	31.32	2135	30.23	
Level 3	4699	16.27	3500	16.05	1199	16.98	
Level 4	3895	13.49	2928	13.42	967	13.69	
Level 5	566	1.96	412	1.89	154	2.18	
Level 6	1082	3.75	801	3.67	281	3.98	
Level 7	1062	3.68	787	3.61	275	3.89	
**CCI score**							< 0.001
0	17,704	61.32	13,526	62.01	4178	59.16	
1	6902	23.90	5069	23.24	1833	25.96	
2	2666	9.23	2006	9.20	660	9.35	
≧ 3	1601	5.54	1210	5.55	391	5.54	
**Other illness**							
Atrial fibrillation							0.345
No	28,663	99.27	21,646	99.24	7017	99.36	
Yes	210	0.73	165	0.76	45	0.64	
Hypertension							0.001
No	21,644	74.96	16,467	75.50	5177	73.31	
Yes	7229	25.04	5344	24.50	1885	26.69	
Lower limb fracture							0.416
No	28,701	99.40	21,676	99.38	7025	99.48	
Yes	172	0.60	135	0.62	37	0.52	
**Hospital level**							< 0.001
Medical centers	13,492	46.73	9823	45.04	3669	51.95	
Regional hospitals	10,162	35.20	7533	34.54	2629	37.23	
District hospitals	3448	11.94	2844	13.04	604	8.55	
clinics	1771	6.13	1611	7.39	160	2.27	
**Hospital ownership**							< 0.001
Public	8201	28.40	5776	26.48	2425	34.34	
Private	20,672	71.60	16,035	73.52	4637	65.66	
**Physician services volume**							< 0.001
Low	3065	10.62	2831	12.98	234	3.31	
High	25,808	89.38	18,980	87.02	6828	96.69	

N = number, y = years, sd = standard deviation, NT dollars = New Taiwan dollars.

CCI is Charlson Comorbidity Index.

### Propensity score matching

Ultimately, 14,124 patients in the conventional drug group and 7,062 patients in the biologic group were matched based on propensity score (Table 2). One hundred and ninety-five patients with VTE, including 143 in the conventional drug group and 52 in the biologic group, were hospitalized (Table 3). An analysis of VTE variables indicated that the incidence of VTE in the biologic group (14.33/10,000 person-years) was higher than that in the conventional drug group (12.61/10,000 person-years). Based on age stratification, both groups had a higher incidence of VTE among patients aged 75–100 (conventional drug group: 40.67/10,000 person-years; biologic group: 45.16/10,000 person-years). When stratified by CCI, both groups had a higher incidence of VTE in patients with CCI > 3 (conventional drug group: 37.89/10,000 person-years; biologic group: 23.55/10,000 person-years). Patients with lower extremity fractures in both groups had a higher incidence of VTE (conventional drug group: 47.34/10,000 person-years; biologic group: 167.08/10,000 person-years).

**Table 2:** Analysis of RA patients using different DMARDs by propensity score matching.

Variables	Total		cDMARD		bDMARD		*p* Value
	N	%	N	%	N	%	
Total number	21,186	100.00	14,124	66.67	7062	33.33	
**Sex**							0.958
Male	4809	22.70	3204	22.68	1605	22.73	
Female	16,377	77.30	10,920	77.32	5457	77.27	
**Age (years)**							1.000
18–34	1835	8.66	1225	8.67	610	8.64	
35–44	2910	13.74	1935	13.70	975	13.81	
45–54	5761	27.19	3844	27.22	1917	27.15	
55–64	6190	29.22	4128	29.23	2062	29.20	
65–74	3257	15.37	2170	15.36	1087	15.39	
75–100	1233	5.82	822	5.82	411	5.82	
Mean age (y ± sd)	54.03 ± 13.34		54.06 ± 13.38		53.95 ± 13.26		0.549
**CCI**							0.999
0	12,537	59.18	8359	59.18	4178	59.16	
1	5492	25.92	3659	25.91	1833	25.96	
2	1988	9.38	1328	9.40	660	9.35	
≧ 3	1169	5.52	778	5.51	391	5.54	
**Other illness**							
**Atrial fibrillation**							0.346
No	21,067	99.44	14,050	99.48	7017	99.36	
Yes	119	0.56	74	0.52	45	0.64	
**Hypertension**							0.729
No	15,564	73.46	10,387	73.54	5177	73.31	
Yes	5622	26.54	3737	26.46	1885	26.69	

N = number, y = years, sd = standard deviation, NT dollars = New Taiwan dollars.

CCI is Charlson Comorbidity Index.

**Table 3:** Risk and related factors of VTE in match RA patients using different DMARDs.

Total number = 21,186															
Variables	cDMARD			bDMARD			Non-DVT		DVTDVT		Log-rank				
	N	Exposed^1^	Incidence^2^	N	Exposed^1^	Incidence^2^	n	%	n	%	*p*-value	aHR^3^	95% CI		*p*-value
VTE	143	113,438.38	12.61	52	36,297.40	14.33	20,991	99.08	195	0.92					
**Biologics**											0.483				
No (ref.)							13,981	98.99	143	1.01		1.00			
Yes							7010	99.26	52	0.74		1.11	0.79	1.55	0.546
**Sex**											0.162				
Female (ref.)	112	89,894.90	12.46	34	28,417.03	11.96	16,231	99.11	146	0.89		1.00			
Male	31	23,543.49	13.17	18	7880.37	22.84	4760	98.98	49	1.02		1.09	0.78	1.51	0.619
**Age (yeas)**											< 0.001				
18–34 (ref.)	5	10,832.14	4.62	2	3459.51	5.78	1828	99.62	7	0.38		1.00			
35–44	7	17,248.77	4.06	2	5314.75	3.76	2901	99.69	9	0.31		0.80	0.30	2.15	0.658
45–54	26	33,382.59	7.79	11	10,444.45	10.53	5724	99.36	37	0.64		1.59	0.70	3.58	0.266
55–64	42	31,769.61	13.22	19	10,378.13	18.31	6129	99.01	61	0.99		2.45	1.11	5.44	0.027
65–74	45	15,779.67	28.52	11	5150.52	21.36	3201	98.28	56	1.72		3.93	1.74	8.89	0.001
75–100	18	4425.61	40.67	7	1550.03	45.16	1208	97.97	25	2.03		5.43	2.24	13.17	< 0.001
Mean age (y ± sd)	60.89 ± 12.70			60.63 ± 11.33											
**Month salary (NT dollars)**											0.418				
≦ 20,008 (ref.)	36	27,848.52	12.93	7	3831.71	18.27	3308	98.72	43	1.28		1.00			
20,009–22,800	48	28,464.85	16.86	14	11,011.54	12.71	5853	98.95	62	1.05		0.99	0.66	1.48	0.967
22,801–28,800	18	20,718.64	8.69	15	8414.64	17.83	4563	99.28	33	0.72		0.76	0.47	1.23	0.266
28,801–36,300	13	10,829.20	12.00	3	4267.68	7.03	2194	99.28	16	0.72		0.86	0.47	1.56	0.610
36,301–45,800	18	12,485.22	14.42	6	4764.83	12.59	2603	99.09	24	0.91		1.05	0.62	1.77	0.865
≧ 45,801	10	13,091.94	7.64	7	4007.01	17.47	2470	99.32	17	0.68		0.74	0.41	1.32	0.302
**Urbanization**											0.018				
Level 1 (ref.)	45	33,940.57	13.26	15	10,749.26	13.95	6263	99.05	60	0.95		1.00			
Level 2	36	36,433.77	9.88	11	10,889.11	10.10	6554	99.29	47	0.71		0.73	0.49	1.07	0.107
Level 3	19	18,140.27	10.47	11	5983.69	18.38	3369	99.12	30	0.88		0.91	0.59	1.42	0.685
Level 4	22	14,888.94	14.78	8	5033.08	15.89	2834	98.95	30	1.05		0.95	0.60	1.50	0.821
Level 5	3	1818.54	16.50	0	819.99	0.00	395	99.25	3	0.75		0.51	0.16	1.65	0.260
Level 6	12	4140.35	28.98	4	1404.72	28.48	788	98.01	16	1.99		1.45	0.81	2.60	0.215
Level 7	6	4075.95	14.72	3	1417.54	21.16	788	98.87	9	1.13		0.88	0.42	1.81	0.719
**CCI score**											< 0.001				
0 (ref.)	60	67,968.05	8.83	24	22,095.12	10.86	12,453	99.33	84	0.67		1.00			
1	41	29,850.78	13.73	18	9406.38	19.14	5433	98.93	59	1.07		1.29	0.92	1.81	0.147
2	21	10,077.87	20.84	6	3097.47	19.37	1961	98.64	27	1.36		1.37	0.87	2.15	0.173
≧ 3	21	5541.68	37.89	4	1698.43	23.55	1144	97.86	25	2.14		2.02	1.26	3.24	0.004
**Other illness**															
Atrial fibrillation											< 0.001				
No (ref.)	140	113,004.99	12.39	51	36,125.57	14.12	20,876	99.09	191	0.91		1.00			
Yes	3	433.39	69.22	1	171.83	58.20	115	96.64	4	3.36		2.04	0.74	5.62	0.167
Hypertension											< 0.001				
No (ref.)	82	86,253.36	9.51	30	27,290.18	10.99	15,452	99.28	112	0.72		1.00			
Yes	61	27,185.02	22.44	22	9007.22	24.42	5539	98.52	83	1.48		1.32	0.97	1.81	0.078
**Lower limb fracture**										< 0.001					
No (ref.)	140	112,804.65	12.41	49	36,117.85	13.57	20,877	99.10	189	0.90		1.00			
Yes	3	633.73	47.34	3	179.56	167.08	114	95.00	6	5.00		3.60	1.58	8.22	0.002
**Hospital level**											0.597				
Clinics (ref.)	11	10,156.09	10.83	1	865.28	11.56	1217	99.02	12	0.98		1.00			
District hospitals	16	16,282.70	9.83	5	3161.70	15.81	2460	99.15	21	0.85		0.80	0.39	1.65	0.552
Regional hospitals	54	37,358.39	14.45	19	13,167.95	14.43	7420	99.03	73	0.97		1.10	0.59	2.06	0.769
Medical centers	62	49,641.20	12.49	27	19,102.47	14.13	9894	99.11	89	0.89		1.09	0.58	2.05	0.799
**Hospital ownership**											0.333				
Public **(ref.)**	30	29,714.69	10.10	19	12,555.41	15.13	6117	99.21	49	0.79		1.00			
Private	113	83,723.69	13.50	33	23,741.99	13.90	14,874	99.03	146	0.97		1.17	0.84	1.63	0.363
**Physician services volume**										0.335					
**Low (ref.)**	16	17,635.97	9.07	4	1270.21	31.49	2117	99.06	20	0.94		1.00			
**High**	127	95,802.41	13.26	48	35,027.19	13.70	18,874	99.08	175	0.92		1.46	0.90	2.37	0.124

N = number, y = years, sd = standard deviation, NT dollars = New Taiwan dollars.

The unit of Exposed is person-years.

Incidence is defined as events per 10,000 person-years.

CCI is Charlson Comorbidity Index.

Log-rank is Log rank test.

aHR is adjusted Hazard Ratio.

### Cox proportional hazards model

As assessed by the Cox proportional hazards model (Table 3), the relative HR for VTE in the biologic group versus that in the conventional drug group was 1.11 (95% CI 0.79–1.55), but did not reach a significant difference (*p *> 0.05). Based on age stratification, the highest relative HR was found in the 75–100 years group (HR: 5.43, 95% CI 2.24–13.17, *p *< 0.05). In the stratified analysis by CCI, the highest relative HR was found in patients with CCI > 3 (HR: 2.20, 95% CI 1.26–3.24, *p *< 0.05). The relative HR for VTEwas 3.60 times higher in patients with lower extremity fractures than in those without (95% CI 1.58–8.02, *p* < 0.05).

## Discussion

In this study, we compared the risk and factors associated with VTE in patients with rheumatoid arthritis using different DMARDs. The results revealed that the incidence of VTE was higher in the biologic group (14.33/10,000 person-years) than in the conventional drug group (12.61/10,000 person-years). The risk of VTE in the biologic group was 1.11 times higher than that of the conventional group, but this did not reach a level of significance.

Some studies have found that the use of TNF-α inhibitors in patients with rheumatoid arthritis reduces inflammation and coagulation markers, as well as decreases the inhibition of fibrinolysis and the incidence of VTE^18,19^. However, several studies have reported cases of VTE after treatment of rheumatoid arthritis or other inflammatory diseases with TNF-α inhibitors^20–24^.

The BSRBR prospective cohort study showed no significant association between TNF-α inhibitors and VTE in patients with rheumatoid arthritis, with an adjusted HR of 0.8 (95% CI 0.5–1.5)^17^. A French study indicated that treatment with TNF-α inhibitors increased the incidence of VTE, accounting for 4.5% of all spontaneously reported adverse reactions to the three TNF-α inhibitors in the database^25^. The findings presented at the 2013 meeting sponsored by the ACR and the ARHP showed that patients with rheumatoid arthritis were at an increased risk of VTE within 180 days of the initiation of biologics (HR: 2.48, 95% CI 1.14–5.40). The PS-decile stratification hazard ratio of VTE associated with biologic DMARDs was 1.83 (95% CI 0.91–3.66) versus nonbiologic DMARDs, but this did not reach significance^16^.

The results of this study were similar to the findings of the previously mentioned US and French studies. The adjusted HR for VTE did not reach a significance; however, given the higher incidence of VTE in the biologic group compared to the conventional drug group, possible clinical implications must be considered.

This study took into account the issue of non-differential misclassification, which may affect the results. Patients of biologic group may remain at risk of developing VTE from conventional DMARDs during the half-life of the drug following termination of treatment. For example, the most widely used MTX has an elimination half-life of 3–10 h and therefore requires a maximum of approximately 55 h for complete elimination. Furthermore, the average interval from cDMARD withdrawal, switching to bDMARD, to VTE was about 924 days based on the Table 4. In this study, the minimum time to develop VTE in the biologic group was found to be 77 days; therefore, this should not affect the results (Table 4).

**Table 4:** Observation time (days) from the initiation of treatment in RA patients with conventional or biologic DMARD to VTE.

Group	N	Mean	SD	*p*-value*	Median	Q1	Q3	Min	Max
Total	195	1675.92	1244.06		1354	736	2492	6	5314
cDMARD	143	1780.20	1327.60	0.023	1433	750	2605	6	5314
bDMARD	52	1389.20	930.50		1082.5	714.5	2315	77	3073

t-test only included cases with VTE (not including those without VTE).

N = number, SD = standard deviation, Min = minimum, Max = maximum.

The unit of measurement in Mean, SD, Median, Q1, Q3, Min and Max is days.

As biologics are expensive, patients may choose to alter their treatment plan due to insurance coverage, and treatment deviations may affect the findings of this study. However, the current regulation for biologics use in Taiwan through national health insurance greatly reduces the possibility of such treatment deviations.

Outpatient follow-up of patients with VTE at a low risk of death (e.g., patients without congestive heart failure or severe hepatic insufficiency) was not included in this study^26^. Previous studies have shown that confirmation of outpatient VTE was less reliable and was not used in comparative studies of rheumatoid arthritis^16,27^. Therefore, despite underestimating the incidence of VTE, this study used patients with inpatient VTE only to improve comparability between treatment groups.

In previous studies, the incidence of VTE has been found to be related to age, fracture type, and chronic disease. The present study confirmed these independent risk factors. In a study from the US, increasing age was associated with a higher risk of VTE when using any conventional DMARD or biologic for therapy^28^. The same results were found in the present study, with incidence increasing with age. The relative hazard of VTE was higher at an older age, and patients aged 55 or older were considered a high-risk group, which could be related to reduced mobility. In addition, several studies have suggested a higher risk of VTE after lower extremity fracture^29–33^. In the present study, the risk of VTE in patients with lower extremity fractures was 3.6 times higher than that in those without, and lower extremity fractures also increased the risk of VTE. Further, previous studies have indicated that the incidence of VTE was associated with some chronic diseases^34–38^. In the present study, the risk of VTE was 2.20 times higher in patients with a CCI greater than 3. Thus, CCI modified the risk of VTE.

The strength of this study lies in the large sample size and the fact that two cohorts were collected simultaneously, which could not be achieved in a clinical trial. Despite the limitations of the observational design, the data from the two cohorts were primarily from medical facilities located throughout Taiwan and the data presented here reflect a real-world experience with DMARDs and biologics for the treatment of rheumatoid arthritis in Taiwan.

Despite great efforts to make adjustments in this study, it should be acknowledged that there may be potential bias caused by unmeasured or unknown variables. As disease severity is a criterion for RA treatment, patients using biologics tend to have more severe rheumatoid arthritis than those using conventional DMARDs^39,40^. A higher disease severity is appropriate in representing a pre-thrombotic state and may relate to reduced patient mobility may lead to venous stasis^38,41–44^. The NHIRD does not provide detailed information of RA severity scale, the data such as disease activity, functional impairment and physical damage was unavailable for the study patients. However, changes in disease severity during the follow-up period did not seem to affect the result much here. Further, some studies have shown that long-term steroid use increased the risk of VTE^45,46^. Since both groups of RA patients had similar conditions during the same time frame, the effect of steroid use on the results of this study would be reduced during the follow-up period.

Due to the limitation of the data contained in the NHIRD, the data related to body mass index and healthy behaviors (such as smoking, alcohol consumption, and exercise) were not available for the study patients; consequently, some important potential confounding factors were not included in the statistical analysis model for discussion. Although treatment and lifestyle changes may benefit from lack of exercise and obesity in RA patients, there is no clear evidence that these factors are related to accelerate atherosclerosis^47^. Besides, there may be a healthy patient effect in this study with RA patients using conventional drugs being excluded from the biologic group if they had VTE, and therefore, RA patients in the biologic group may have been in better health than those in the conventional drug group. This could have a biased effect on the study results. Additionally, this study population was a predominantly Asian population in Taiwan, and therefore, it may be inappropriate to apply the findings to other ethnic groups or regions with different rates of VTE. Because the incidence of VTE is very low and the total number of events relatively small, the confidence interval is wide, and the result lacks power. This is a study limitation, as type 2 error cannot be ruled out.

## Conclusion

In summary, this study found no evidence that biologic therapy was associated with an increased risk of VTE in patients with rheumatoid arthritis, and no significant differences in risk were observed between the use of conventional DMARDs and biologics.

## Methods

The study was a retrospective national population-based cohort study. We extracted the claims data from the National Health Insurance Research Database (NHIRD) and Registry for Catastrophic Illness Patient Database (RCIPD) provided by the Ministry of Health and Welfare. In Taiwan, The National Health Insurance (NHI) program covers 99.9% of the population, and under the insurance program, copayments for RA patients is waived^48^. The NHI administration has also included 93% of Taiwan’s health services organizations as NHI-contracted health care providers as of the end of 2014^48^. Diagnoses of RA were coded according to the International Classification of Diseases, Ninth Revision, Clinical Modification (ICD-9-CM). Several previous studies had demonstrated the high accuracy and validity of ICD-9 diagnosis of major diseases listed in the NHIRD^49,50^. This study was approved by the Institutional Review Board of Taichung Veterans General Hospital (TCVGH-IRB No.: CE20255B) and was conducted in accordance with the Helsinki Declaration. Identification information of all patients was omitted prior to analysis. Since the patient identifications in the National Health Insurance Research Database have been de-identified by the Taiwan Ministry of Health and Welfare, the informed consent was waived by the Research Ethnics Committee of Taichung Veterans General Hospital.

Since biologic agents were available for treatment of RA in Taiwan in 2003, we identified all patients who had been newly approved for Catastrophic Illness Card of rheumatoid arthritis from 2003 to 2016, and the follow-up end point was set as December 31, 2017. The accuracy of diagnosis was validated based on ICD codes (ICD-9-CM 714.0–714.9) and inclusion in the RCIPD. The exclusion criteria for this study were as follows: (1) had a total hip arthroplasty or total knee arthroplasty (2) VTE and PE prior to the index date owing to a high recurrence rate of 7–14%^6^; (3) other major injuries or illness prior to the index date, which contains 30 categories of severe illness or injury defined by the NHI; (4) under 18 years of age; and (5) incomplete data in the NHIRD and TCRD. The exclusion period also included the years 2000–2002.

The two groups using DMARDs were defined as (1) a conventional DMARD group (cDMARD, including Methotrexate, Hydroxychloroquine, Sulfasalazine, Leflunomide, D-penicillamine, Azathioprine, Mycophenolate, Cyclosporine, Tafacitinib) and (2) a biologic DMARD group (bDMARD, including Etanercept, Adalimumab, Golimumab, Rituximab, Abatacept, Tocilizumab, Infliximab, Certolizumab). Subjects in the conventional drug group were not allowed to use biologics concurrently; however, they were allowed to use other DMARDs concurrently. In Taiwan, for the treatment with biologics should be through national health insurance requiring a detailed evaluation of the RA patient to meet the criteria including the 28 Disease Activity Score must be greater than 5.1 and failed other DMARDs therapy. Therefore, almost all patients in the bDMARD group using biologics were after cDMARDs withdrawal. And in some cases, it may switch to other bDMARDs, but not to other cDMARDs. Biologics use was defined in this study as any exposure to any biologic agent during the observation period. In both groups, the index date was defined as the date of first treatment (either with a conventional drug or biologics, dependent on group) until the date of first VTE, last follow-up, death, or end of observation period.

In this study, VTE was defined as the presence of inpatient VTE diagnostic codes (including DVT or PE) according to the discharge diagnosis protocol. The diagnostic codes for VTE were DVT (DVT: ICD-9-CM codes: 451.11, 451.19, 451.2x, 453.8x, and 453.9x) or PE (PE: ICD-9-CM codes: 415.11 and 415.19). The discharge notes and medical reimbursement of DVT and PE should be scrutinized by peer review.

Demographic data including age at the date of diagnosis confirmation were documented. The urbanization level ranged from highly developed urban cities (level 1) to remote districts (level 7)^51^. The degrees of comorbidity were categorized into three levels according to the Charlson comorbidity index (CCI) modified by Deyo^52^. The CCI score containing 19 medical issues was a useful method for evaluating medical comorbidities. Other variables included atrial fibrillation, hypertension, lower limb fracture, patients’ monthly salary, hospital level (medical centers, regional hospitals, district hospitals, and clinics), and the annual service volume of primary care physicians (low and high).

All statistical analyses were performed using SAS software, version 9.2 (SAS Institute Inc., Cary, NC, USA), and statistical significance was defined as *p* < 0.05. A chi-square test was applied to compare the distribution of each variable between the two groups at the beginning of the study (baseline characteristics). When we conducted the propensity score matching (PSM) method, the dependent variable was patients using conventional DMARD or biologic DMARD. The independent variables which were used in the logistic regression model included sex, age, severity of comorbidity (CCI), atrial fibrillation, and hypertension at the index date of the first biologic treatment. An SAS matching macro, %OneToManyMTCH, was used for the PSM. The PSM was performed by using the greedy nearest neighbor matching by digit without replacement to form a subject matching set with a 2:1 matching ratio on the propensity score. The algorithm performed the “best” match first, followed by the “next best” match in hierarchical sequence, until no more matches could be made. Best matches were those with the highest digit match on propensity score. Each control is selected at most once. The final matched-pair samples contain both closely matched individual pairs and balanced control and case group. Approximately 73% of total study population was matched in the final matched sample. Subsequently, a chi-square test was used to compare the incidence of VTE among patients with rheumatoid arthritis treated with different drug regimens and the Cox proportional hazards model was applied to assess the relative hazard and factors of VTE after controlling for other factors. The relative hazard was also shown using the total events of VTE, the incidence of VTE (defined as events per 10,000 person-years), the adjusted hazard ratio (HR), and 95% confidence interval (CI).

## Data Availability

Regarding the data availability, data were obtained from the National Health Insurance Research Database published by the Ministry of Health and Welfare, Taiwan. Due to legal restrictions imposed by the Taiwan government related to the Personal Information Protection Act, the database cannot be made publicly available. We confirm that the data used in our study can be used by any future researchers and we did not receive special privileges from the Ministry of Health and Welfare, Taiwan. All researchers can apply for using the databases to conduct their studies in the Science Center of the Ministry of Health and Welfare (http://www.mohw.gov.tw/EN/Ministry/Index.aspx). Any raw data are not allowed to be brought out from the Health and Welfare Data Science Center. The restrictions prohibited the authors from making the minimal data set publicly available. This study used anonymized secondary data retrieved from the Taiwan Cancer Registry Database and Taiwan’s National Health Insurance Research Database; consequently, the requirement for informed consent was waived by the ethics committee.

## Code availability

Due to privacy and proprietary natures, no code in this study can be made available.
